# Let’s Go Bananas: Beyond Bounding Box Representations for Fisheye Camera-Based Object Detection in Autonomous Driving †

**DOI:** 10.3390/s25123735

**Published:** 2025-06-14

**Authors:** Senthil Yogamani, Ganesh Sistu, Patrick Denny, Jane Courtney

**Affiliations:** 1 School of Electrical & Electronic Engineering, Technological University Dublin, D07 ADY7 Dublin, Ireland; jane.courtney@tudublin.ie; 2 D2ICE Research Centre, University of Limerick, V94 T9PX Limerick, Ireland; ganesh.sistu@ul.ie (G.S.); patrick.denny@ul.ie (P.D.); 3 Department of Computer Science & Information Systems (CSIS), University of Limerick, V94 T9PX Limerick, Ireland

**Keywords:** automated driving, object detection, surround view cameras, fisheye cameras

## Abstract

Object detection is a mature problem in autonomous driving, with pedestrian detection being one of the first commercially deployed algorithms. It has been extensively studied in the literature. However, object detection is relatively less explored for fisheye cameras used for surround-view near-field sensing. The standard bounding-box representation fails in fisheye cameras due to heavy radial distortion, particularly in the periphery. In this paper, a generic object detection framework is implemented using the base YOLO (You Only Look Once) detector to systematically explore various object representations using the public WoodScape dataset. First, we implement basic representations, namely the standard bounding box, the oriented bounding box, and the ellipse. Secondly, we implement a generic polygon and propose a novel curvature-adaptive polygon, which obtains an improvement of 3 mAP (mean average precision) points. A polygon is expensive to annotate and complex to use in downstream tasks; thus, it is not practical to use it in real-world applications. However, we utilize it to demonstrate that the accuracy gap between the polygon and the bounding box representation is very high due to strong distortion in fisheye cameras. This motivates the design of a distortion-aware optimal representation of the bounding box for fisheye images, which tend to be banana-shaped near the periphery. We derive a novel representation called a curved box and improve it further by leveraging vanishing-point constraints. The proposed curved box representations outperform the bounding box by 3 mAP points and the oriented bounding box by 1.6 mAP points. In addition, the camera geometry tensor is formulated to provide adaptation to non-linear fisheye camera distortion characteristics and improves the performance further by 1.4 mAP points.

## 1. Introduction

**Figure 1:**
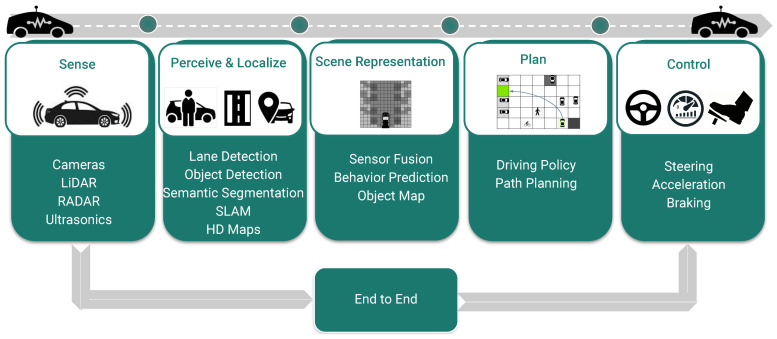
Components of a typical Autonomous Driving Pipeline.

When an autonomous vehicle moves from source to destination, a navigator like Google Maps or HD (high definition) Maps generates a high-level route. This route is made up of a series of connected nodes at finite distances. The vehicle moves from one node to another repetitively until it reaches the destination. Maneuver occurs in a five-stage process: sensing, perception and localization, scene representation, planning, and controlling (illustrated in Figure 1) [1].

In the sensing stage, the vehicle collects information about the surroundings via sensors like cameras, LiDAR, RADAR, and ultrasonics. Perception involves the extraction of useful information from the raw data, and it is the most studied module in the literature. However, there is limited literature on perception tasks on fisheye cameras like depth estimation [2], moving object detection [3], and object detection [4]. Fusion combines the perception from different sensors, primarily cameras and LiDAR [5]. Localization is the vehicle’s ability to precisely know its position in the real world at decimeter accuracy [6]. In simple words, perception answers what is around the vehicle, and localization answers where the vehicle is precisely. Path planning algorithms [7] make use of this related information to define a path to navigate from one node to another. While defining the path, the algorithms use different driving policies like safety, rules of driving, road conditions, and pleasant ride experience for the passengers in the car [8]. These digital instructions from the algorithms are converted into the vehicle’s physical movement via the controlling unit [9].

In the autonomous driving pipeline, perception is a computationally expensive and sophisticated block, and efforts to build unified models for all perception-related tasks are an active area of research [10]. Though there is a considerable debate on what sensors are needed for this task, cameras are considered an essential sensor for any perception pipeline. Visual perception is the task of perceiving the information around the vehicle via cameras. A modern automated vehicle consists of anywhere between 4 and 20 cameras of different fields of view (FoV) performing different tasks. Visual perception can be described as a combination of recognition, reconstruction, and relocalization. Recognition is knowing what is around the vehicle and involves tasks like detection and segmentation. Reconstruction consists of depth estimation and motion estimation to know where the objects are in the 3D world. Relocalization knows where the ego vehicle is in the world. It involves pose estimation and SLAM (simultaneous localization and mapping) [6]. Other than this, perception also involves lesser-known tasks like trailer angle estimation and measuring sun glare on the lens.

In this work, we aim to design an object detection system that can be deployed in real time on commercially available embedded systems. Thus, we do not use advanced architectures like transformers and focus on a simpler, efficient architecture. Recently, demand for low-power SoC (system-on-chip)-based automated vehicles has increased significantly as features like pedestrian detection, emergency braking, and lane keep assist started to attract more consumers. The choice of SoC is based on the criteria of performance (Tera operations per second (TOPS), utilization, bandwidth), cost, power consumption, heat dissipation, high- to low-end scalability, and programmability. The SoC choice provides the computational bounds in the design of algorithms. The progress in convolutional neural networks (CNNs) has also led the hardware manufacturers to include their custom hardware computing units to provide a high throughput of over 10 TOPS targeting Level-3 automation. But Level-2 automation systems still rely on computing units less than 2 TOPS. In [11], authors developed a multi-task learning algorithm for hardware with 1 TOPS of computing power, consuming less than 10 watts of power.

There is very limited work on fisheye camera object detection for autonomous driving. The main issue is the lack of public datasets, which we resolved by creating the first fisheye camera dataset called WoodScape [12] and baseline object detection models [13] in our previous work. In this paper, we provide a comprehensive study of fisheye object detection with several novel methods building upon our previous work as an expanded journal paper. Our main contributions include

- Implementation of an extensible YOLO-based framework facilitating comparison of various object representations for fisheye object detection.
- Design of novel representations for fisheye images, including the curved bounding box, vanishing point-based curved bounding box, and adaptive step polygon.
- Design of novel camera tensor representation of radial distortion to enable adaptation of the model to specific camera distortions.
- Ablation study of effect of undistortion, encoder type and temporal models.

## 2. Related Work

### 2.1. Object Detection

Object detection is the first and foremost problem in visual perception, which involves the recognition and localization of the objects in the image. It has use cases like emergency braking and collision avoidance, etc. Hence, object detection models’ performance directly influences the success and failure of autonomous driving systems. A simple and efficient representation of objects in images is bounding box representation. State-of-the-art methods for object detection based on deep learning can be broadly classified into two types, namely single-stage and two-stage detectors.

#### 2.1.1. Two Stage Object Detection

In two-stage approaches, object detection is split into two tasks: (i) Extraction of regions of interest (ROIs) and encoding them as features (ii) Regressing for bounding boxes in these ROIs using encoded features. A common practice is to have a high recall in the first stage to ensure all possible objects, like patterns, go through the second stage. RCNN (region-based convolutional neural networks) [14] was the first to use this approach. In the RCNN first stage, a selective search algorithm is used to propose ROIs, followed by CNN feature extraction. In the second stage, an SVM is trained to classify the objects based on the CNN features. Unlike RCNN, which extracts CNN features separately on each ROI, Fast-RCNN [15] processes the whole image. So the CNN feature extraction is performed only once per image. It has introduced a 25× speed in the inference stage compared to RCNN. Also, Fast-RCNN has replaced SVM (support vector machines) with a linear classifier and introduced a linear regressor for bounding box fine-tuning. This moved the Fast-RCNN a step closer to the end differentiable training strategy. Both Fast-RCNN [15] and SPP-net [16] improved RCNN [14] by extracting RoIs from the feature maps. SPP-net introduced a spatial pyramid pooling (SPP) layer to handle images of arbitrary sizes and aspect ratios. It applies an SPP layer over the feature maps generated from convolution layers and outputs fixed-length vectors required for fully connected layers. It eliminates fixed-size input constraints and can be used in any CNN-based classification model. However, Fast-RCNN and SPPnet are not end-to-end trainable as they depend on the region proposal approach. Faster-RCNN [17] solved this limitation by introducing Region Proposal Network (RPN), which made end-to-end training possible. RPN generates RoIs by regressing a set of reference boxes, known as anchor boxes. This introduced two streams for object detection, i.e., a common encoder and two decoders. The efficiency of Faster-RCNN is further improved by RFCN (Region-based Fully Convolutional Networks) [18], which replaces fully connected layers with fully convolutional layers.

#### 2.1.2. Single Stage Object Detection

These approaches eliminate the RoI extraction stage and carry out classification and regression of bounding boxes directly on CNN feature maps, and hence a single-state encoder-decoder style network performs localization and classification tasks. Overfeat [19] proposed a unified framework to perform two tasks: classification and localization using a multi-scale, sliding window approach. YOLO [20] divides the input image into grids and predicts bounding boxes directly by regression and classification at each grid. This soon became a de facto style for single-state real-time object detection on low-power hardware like mobile phones and Level 3 autonomous driving engines. YOLO9000 (YOLOv2) [21] improved the performance by introducing batch normalization and replacing fully connected layers of YOLOv1 with anchor boxes for bounding box prediction. Anchor boxes are computed over the dataset, representing the average variation of height and width of the objects in the dataset. Instead of directly regressing for object width and height, YOLOv2 predicts offsets from a predetermined set of anchors with particular height-width ratios. YOLOv3 [22], a faster and more accurate object detector than previous versions, uses Darknet-53 as its feature extraction backbone. YOLOv3 can detect small objects with a multi-scale prediction approach, a significant drawback in earlier versions.

Single Shot Multibox Detection (SSD) [23] places dense anchor boxes over the input image and extracts feature maps from multiple scales. It then classifies and regresses the bounding boxes relative to anchor boxes. DSSD (Deconvolutional Single Shot Detector) [24] replaced the VGG (Visual Geometry Group) network of SSD with Residual-101. It is then augmented with a deconvolution module to integrate feature maps from the early stage with the deconvolution layers. It outperforms the SSD in detecting small objects. MDSSD (Multi-scale Deconvolutional Single Shot Detector) [25] further extends DSSD with fusion blocks to handle feature maps at different scales.

RetinaNet [26] introduced focal loss to address foreground and background class imbalance during training. It matches or surpasses the accuracy of state-of-the-art two-stage detectors while running at faster speeds. The architecture shares ‘anchors’ from RPN and builds a single fully convolutional network (FCN) with feature pyramid network (FPN) on top of the ResNet backbone.

### 2.2. Instance Segmentation

Instance segmentation involves predicting both object bounding boxes and pixel-level object masks.

#### 2.2.1. Two Stage Instance Segmentation

Intuitively, instance segmentation can be modeled as a bounding box detection followed by a binary segmentation within the box. This paradigm is referred to as ‘Detection then Segmentation.’ Models following this approach often achieve a state-of-the-art performance but are quite slow to adapt to real-time applications. MaskRCNN [27] adapted this approach by using FasterRCNN for bounding box detection and an additional decoder for object mask segmentation. Here segmentation is performed as a binary classification to differentiate object pixels from the background or other object pixels. Multi-task Network Cascade (MNC) [28] uses a similar approach to MaskRCNN. It uses RPN for box proposals, followed by class-agnostic instance generation on the proposed regions and finally categorical classification of these mask instances. During the inference time on a 12 GB, 7 TFLOPs NVIDIA M40 GPU, MaskRCNN reported a 6 FPS run time. Today even the Level-5 autonomous vehicles use only 1.3 to 2 TFLOPs computing engines for running the complete deep learning stack, making a state-of-the-art two-stage approach far from reality for L3 automated vehicles. This led to a recent trend of simplistic single-stage object detection style instance segmentation techniques like PolarMask [29] YOLOACT (You Only Look At CoefficienTs) [30], and PolyYOLO [31].

#### 2.2.2. Single Stage Instance Segmentation

YOLOACT [30] uses a single encoder dual parallel decoder style architecture for instance-level image segmentation. The encoder is the same as the RetinaNet backbone, i.e., Feature Pyramid Network with Resnet101 [32]. The first decoder generates a set of *k* prototype masks at image resolution. These masks do not depend on any single object class. However, these masks represent instance masks of an object when multiplied with the correct set of coefficients. The second decoder is a standard bounding box decoder with extra computation to predict mask coefficients for each object instance. Instance masks for objects are generated as a linear combination of prototype masks and mask coefficients. Though YOLOACT performance is lower than MaskRCNN, it is 5× faster in run time.

#### 2.2.3. Polygon Instance Segmentation

PolarMask [29] and PolyYOLO [31] regress for contour boundaries in polar space. It is hence removing computational overheads of an extra decoder and segmentation of pixels at the image level. Other approaches to instance segmentation range from clustering of instance embedding [33,34] to prediction of instance centers using offset regression [35]. These methods appear intuitively designed but are lagging in terms of accuracy and computational efficiency. The major drawback of these methods is the usage of compute-intensive clustering methods like OPTICS [36] and DBSCAN [37].

### 2.3. Object Detection on Fisheye Cameras

There is relatively less work on object detection for fisheye or closely related omnidirectional cameras [38]. One of the main issues is the lack of a useful dataset, particularly for autonomous driving scenarios. The recent fisheye object detection paper FisheyeDet [39] emphasizes the lack of a useful dataset, and they create a simulated fisheye dataset by applying distortions to the Pascal VOC dataset [40]. FisheyeDet makes use of a 4-sided polygon representation aided by distortion shape matching. SphereNet [41] and its variants [42,43,44] formulate CNNs on spherical surfaces. However, fisheye images do not follow spherical projection models, as seen by non-uniform distortion in horizontal and vertical directions.

## 3. Challenges in Object Detection on Fisheye Cameras

**Figure 2:**
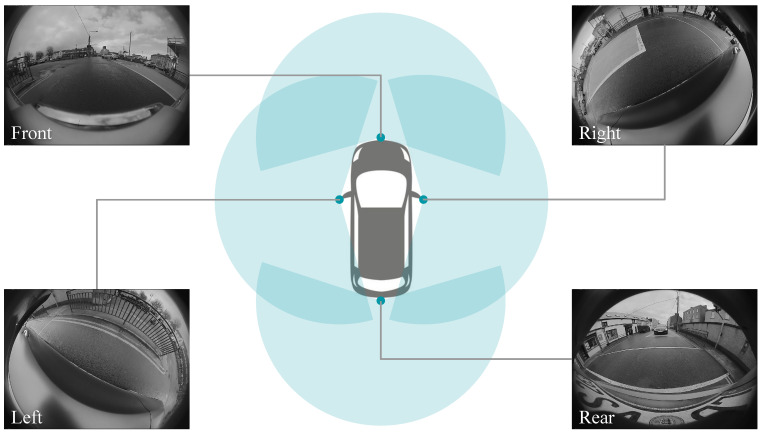
Surround-view camera network images showing near field sensing and wide field of view.

Fisheye cameras make use of non-linear mapping to generate a large field of view. With just four surround-view fisheye cameras, we can achieve a dense 360° near-field perception, making them suitable for automated parking, low-speed maneuvering, and emergency braking. A commercial fisheye camera usually has a 190° horizontal field of view, as shown in Figure 2. It is usually available from 2 MP to 20 MP resolution. However, this advantage comes at the cost of non-linear distortions in the image. Objects at different angles from the project center look quite different, making the object detection a challenge.

**Figure 3:**
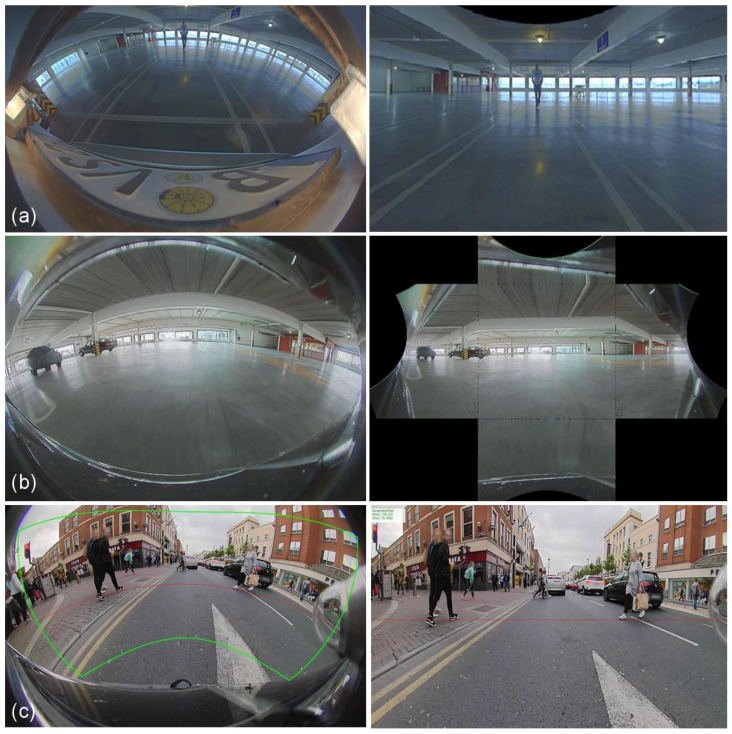
Undistorting the fisheye image: (**a**) Rectilinear correction; (**b**) Piecewise linear correction; (**c**) Cylindrical correction. **Left**: raw image; **Right**: undistorted image. Green curves in (**c**) denote the region which is mapped to an undistorted image and red curve becomes a straight line demonstration removal of distortion.

A common practice is to rectify distortions in the image by a 4th-order polynomial model or unified camera model [45]. The fact is, there is no ideal projection or correction. These corrections are application-driven, and every correction technique has its disadvantages (Figure 3). Rectilinear correction suffers from loss of field of view (FoV) and sampling issues, piece-wise linear with artifacts at transition areas and massive bleeding in the image, and cylindrical as a quasi-linear correction, offers a practical trade-off. Another overhead is extra computational resources needed for correction, as the visual perception pipeline usually has different algorithms demanding different view projections. Though look-up tables (LUTs) make this correction process accelerated, LUTs rely on online calibration that needs to be generated every time there is a change in the online calibration.

Despite these disadvantages, image correction is encouraged due to the limitations of the non- or early deep learning object recognition and segmentation algorithms. With a push in deep neural networks, this trend is slowly changing. Modern CNN-based object detection algorithms like YOLO and FasterRCNN can detect objects on raw fisheye images, and the main issue with object detection on raw fisheye images is representation of objects as bounding boxes.

### 3.1. Bounding Boxes on Fisheye

Objects undergo significant deformation due to radial distortion in fisheye images, causing bounding box representations to fail in many practical scenarios [46]. Here are two scenarios where the correct representation of objects is as important as the detection.

#### 3.1.1. Pedestrian Localization Issue

**Figure 4:**
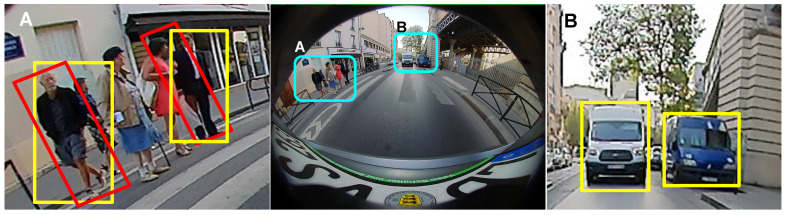
Illustration of examples to show that bounding box is not a good representation on wide-angle fisheye cameras. Patches (**A**,**B**) in the center front camera image are zoomed into left and right images, respectively. As the patch (**B**) is in the center of the fisheye image, which has less distortion, bounding box shown in yellow is sufficient. However, the patch (**A**), which is closer to periphery, suffers from radial distortion, and the yellow bounding box is not a good representation. The red-oriented bounding box is a better representation.

In Figure 4B, vehicles are near the center region of the image, and hence the lower part of the bounding boxes represents the object intersection with the road quite well. However, in Figure 4A, standard bounding boxes in yellow color are not good enough to represent the object road intersection. The common idea is to orient the boxes as shown in red color in Figure 4A. In the case of the person on the left side, this orientation concept works. As the box with optimal orientation is also a box with optimal IoU with the ground truth. However, in the case of the person in a black suit, the optimally oriented box is not the optimal IoU (Intersection over Union). So simple orientation works in some cases, but it does not solve the problem. 3D boxes work, but both annotating and inferring a 3D box is a noisy process for small objects.

#### 3.1.2. Missing Parking Spot

**Figure 5:**
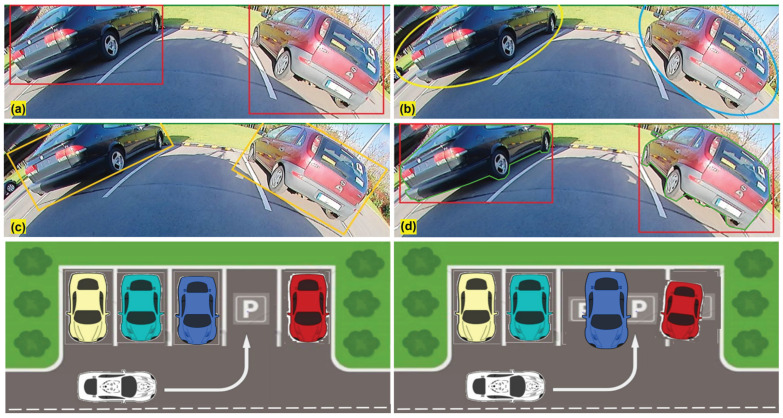
The main use case of the surround view fisheye camera is automated parking. Top: Object detection using bounding box (**a**), ellipse (**b**), oriented box (**c**), and polygon (**d**). Bounding box in (**a**) is overlaid in (**d**) to illustrate the difference. Bottom: Environmental map (used for path planning in top view) illustrating the correct parking slot estimation corresponding to (**d**) on the left and incorrect parking lot estimation on the right corresponding to (**a**).

A correctly detected but improperly represented object can result in failure cases like missing a parking spot or in non-optimal path planning. Figure 5 shows an automated car maneuvering to a parking slot between the two cars. Two cars were detected by bounding box, oriented box, ellipse, and polygon object detection algorithms. However, only in the instance segmentation case are objects located correctly outside the free parking spot. In the remaining cases, objects appear to be present within the parking spot, causing the maneuver mapping to falsely indicate the spot as occupied (Figure 5, bottom row images). This shows that the detection of objects is as important as correct representation in fisheye-based visual navigation systems.

A full-fledged solution to this problem is instance segmentation, but most state-of-the-art algorithms like MaskRCNN demand higher computing powers and are unrealistic to work on low-power hardware that is generally used in Level 2 and Level 3 autonomous vehicles. Hence, there is a need for memory- and computation-efficient models. It encouraged us to develop FisheyeDetNet, a single network to perform object detection and instance segmentation to deploy on low-power hardware accelerators. It is an efficient, small-footprint network that uses ResNet18 as a backbone and YOLO-style head for polygon-based instance segmentation.

## 4. Proposed Method

### 4.1. High Level Architecture

**Figure 6:**
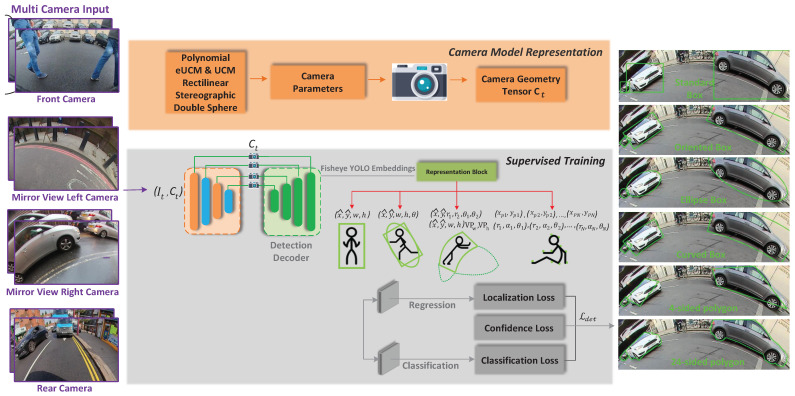
FisheyeDetNet: Proposed object detection network architecture and comparison between different representations. The representation block is designed to be generic for any representation. Within the representation block, we show four options, namely (1) bounding box [represented by center ($\hat{x},\hat{y}$), width (w), and height (h) of the 2D box], (2) oriented bounding box/ellipse [adds an orientation angle (θ) to 2D box representation], and (3) curved bounding box [parameterized by circular curves with a center, two radii ($r_{1},r_{2}$) for inner and outer edges of the circle and two angles ($\theta_{1},\theta_{2}$) for start and finish of the arc. It is simplified by dotted curves using vanishing points in width ($VP_{w}$) and height direction ($VP_{h}$). Refer to Section 4.4 for more details]. (4) Generalized polygon [non-uniformly sampled points are shown on the stick figure either using Cartesian coordinates (x,y) or polar coorodinates (*r*, θ, α)]. Representations (1) to (4) are drawn in fluorescent green from left to right below the red arrows from the representation block.

Figure 6 illustrates the proposed network architecture whose goal is to systematically explore novel representations for fisheye camera object detection. We use the standard encoder-decoder architecture, which is commonly used for dense prediction tasks. We also adopt the Siamese (twin network) approach to leverage temporal cues. Here we concatenate the source and target frame features and pass the fused encoded features to the decoder. As the weights are shared in the Siamese encoder, the previous frame’s encoder can be saved and reused instead of recomputing.

We adapt PolyYOLO [31] into a generic framework called FisheyeDet where a generic representation block is used to implement different output representations. Instead of using sparse polygon points and single-scale predictions like PolyYOLO, we use dense polygon annotations and multi-scale predictions. Instead of a computationally expensive backbone like PolarMask, we employed lightweight ResNet-18 as our encoder to facilitate real-time deployment on an embedded system. These changes enabled us to develop a small-footprint instance segmentation model with just 13 M parameters. As there is no heavy encoder backbone or feature map upscaling to image level and segmentation at the pixel level, our model is quite suitable for real-time applications like object detection on Level-3 automotive ECUs (electronic control unit).

Similar to YOLO, object detection is performed at multiple scales. For each grid in each scale, object width ($\hat{w}$), height ($\hat{h}$), object center coordinates ($\hat{x}$, $\hat{y}$), and object class are inferred. Finally, a non-maximum suppression is used to filter out the low-confidence detections. Instead of using $L_{2}$ loss for categorical and objectness classification, we used standard categorical cross-entropy and binary entropy losses, respectively. To target efficient real-time performance, we start with a simple ResNet18 encoder and then extend to sophisticated self-attention encoders (SAN). We design our encoder by incorporating vector attention-based pairwise and patchwise self-attention encoders from [47]. These networks efficiently adapt the weights across both spatial dimensions and channels.

#### Camera Geometry Tensor

We derived a novel camera geometry tensor in our previous work, SVDistNet [45], for depth estimation. In this work, we integrate this camera geometry tensor for object detection as a mechanism to provide fisheye camera distortion properties as an inductive bias to the network. The camera geometry tensor $C_{t}$ is applied in the encoder-decoder cross connections, as shown in Figure 6. In the case of SAN encoders, it is also included in each self-attention stage in addition to every skip-connection in ResNet architecture.

The camera geometry tensor $C_{t}$ is formulated in a three-step process: For efficient training, the pixel-wise coordinates and angle of incidence maps are pre-computed. The normalized coordinates per pixel are used for these channels by incorporating information from the camera calibration. We concatenate these tensors and represent them by $C_{t}$ and pass it along with the input features to our SAN *pairwise* and *patchwise* operation modules. It comprises six channels in addition to the existing decoder channel inputs. The proposed approach can in principle be applied to any fisheye projection model of choice explained in [48]. The different maps included in our shared self-attention encoder are computed using the camera intrinsic parameters, where the distortion coefficients $a_{1},a_{2},a_{3},a_{4}$ are used to create the angle of incidence maps $\left(a_{x},a_{y}\right)$, $c_{x},c_{y}$ are used to compute the principal point coordinate maps $\left(cc_{x},cc_{y}\right)$, and the camera’s sensor dimensions (width *w* and height *h*) are utilized to formulate the normalized coordinate maps.

***Centered Coordinates (cc)*** Principal point position information is fed into the SAN’s *pairwise* and *patchwise* operation modules by including the $cc_{x}$ and $cc_{y}$ coordinate channels centered at (0,0). We concatenate $cc_{x}$ and $cc_{y}$ by resizing them using bilinear interpolation to match the input feature size. We formulate $cc_{x}$ and $cc_{y}$ channels as

(1) $$cc_{x}=\left(\begin{array}{c} 0-c_{x}\\ 1-c_{x}\\ \vdots \\ w-c_{x} \end{array}\right)\cdot {\left(\begin{array}{c} 1\\ 1\\ \vdots \\ 1 \end{array}\right)}_{(h+1)\times 1}^{⊺},cc_{y}={\left(\begin{array}{c} 1\\ 1\\ \vdots \\ 1 \end{array}\right)}_{(w+1)\times 1}\cdot {\left(\begin{array}{c} 0-c_{y}\\ 1-c_{y}\\ \vdots \\ h-c_{y} \end{array}\right)}^{⊺}$$

***Angle of Incidence Maps ($a_{x},a_{y}$)*** For the pinhole (rectilinear) camera model, the horizontal and vertical angle of incidence maps are calculated from the cc maps using the camera’s focal length *f*: $a_{ch}\left[i,j\right]=\arctan\left(cc_{ch}\left[i,j\right]/f\right)$, where ch can be *x* or *y* (see Equation (1)). For the different fisheye camera models, the angle of incidence maps can analogously be deduced by taking the inverse of the radial distortion functions r(θ) explained in [48]. Specifically, for the polynomial model used in this paper, the angle of incidence θ is formulated by calculating the 4th order polynomial roots of $r\left(\theta \right)=\sqrt{x_{I}^{2}+y_{I}^{2}}=a_{1}\theta +a_{2}\theta^{2}+a_{3}\theta^{3}+a_{4}\theta^{4}$ through a numerical method. We store the pre-calculated roots in a lookup table for all pixel coordinates to achieve training efficiency and create the $a_{x}$ and $a_{y}$ maps by setting $x_{I}=cc_{x}\left[i,j\right],y_{I}=0$ and $x_{I}=0,y_{I}=cc_{y}\left[i,j\right]$, respectively.

***Normalized Coordinates (nc)*** Additionally, we add two channels of normalized coordinates [49,50] whose values vary linearly between −1 and 1 with respect to the image coordinates. The channels are independent of the camera sensor’s properties and characterize the spatial extent of the content in feature space in each direction (e.g., a value of the $\hat{x}$ channel close to 1 indicates that the feature vector at this location is close to the right border).

### 4.2. Basic Object Representations

#### 4.2.1. Bounding Box

We use the standard bounding box representation as the main baseline using object width ($\hat{w}$), height ($\hat{h}$), and object center coordinates ($\hat{x}$, $\hat{y}$). We illustrate the modified YOLO loss as a combination of sub-losses so that it can be extended for other variants.

(2) $$L_{xy}=\lambda_{coord}\sum_{i=0}^{S^{2}}\sum_{j=0}^{B}l_{ij}^{obj}\left[{\left(x_{i}-{\hat{x}}_{i}\right)}^{2}+{\left(y_{i}-{\hat{y}}_{i}\right)}^{2}\right]$$

(3) $$L_{wh}=\lambda_{coord}\sum_{i=0}^{S^{2}}\sum_{j=0}^{B}l_{ij}^{obj}\left[{\left(\sqrt{w_{i}}-\sqrt{{\hat{w}}_{i}}\right)}^{2}+{\left(\sqrt{h_{i}}-\sqrt{{\hat{h}}_{i}}\right)}^{2}\right]$$

(4) $$\begin{array}{cc} L_{obj} & =-\sum_{i=0}^{S^{2}}\sum_{j=0}^{B}C_{i}log\left({\hat{C}}_{i}\right) \end{array}$$

(5) $$\begin{array}{cc} L_{class} & =-\sum_{i=0}^{S^{2}}\sum_{j=0}^{B}l_{ij}^{obj}\sum_{c=classes}c_{i,j}log\left(p\left(\hat{c_{i,j}}\right)\right) \end{array}$$

(6) $$\begin{array}{cc} L_{total} & =L_{xy}+L_{wh}+L_{obj}+L_{class} \end{array}$$

where height and width are predicted as offsets from pre-computed anchor boxes. B refers to the number of bounding boxes, S refers to the grid size, $\lambda_{coord}$ refers to weighting factor of coordinate losses. $C_{i}$ refers to the object class in ground truth, and ${\hat{C}}_{i}$ refers to the predicted class. In $L_{class}$, the i,j suffix denotes the *j*th bounding box predictor in cell *i*, and $l_{ij}$ indicates whether there is an object or not. Please refer to YOLO [20] for more details of these terms.

(7) $$\begin{array}{cc} \hat{w} & =a_{w}*e^{fw} \end{array}$$

(8) $$\begin{array}{cc} \hat{h} & =a_{h}*e^{fh} \end{array}$$

(9) $$\begin{array}{cc} \hat{x} & =g_{x}+f_{x} \end{array}$$

(10) $$\begin{array}{cc} \hat{h} & =g_{y}*f_{y} \end{array}$$

where $a_{w}$ and $a_{h}$ are anchor box width and height. $f_{w}$, $f_{h}$, $f_{x}$, $f_{y}$ are the outputs from the last layer of the network at grid location $g_{x}$, $g_{y}$.

#### 4.2.2. Oriented Bounding Box

The oriented bounding box is a simple extension of the standard bounding box to handle tilted objects that are not axis-aligned to the standard x and y axes. In this representation, along with the regular box information ($\hat{w}$, $\hat{h}$, $\hat{x}$, $\hat{y}$), the orientation of the box $\hat{\theta }$ is also regressed. Orientation ground truth range (−180 to +180°) is normalized between −1 and +1. The loss function is the same as the regular box loss but with an additional term for orientation loss.

(11) $$\begin{array}{cc} L_{orn} & =\sum_{i=0}^{S^{2}}\sum_{j=0}^{B}l_{ij}^{obj}{\left[\theta_{i}-{\hat{\theta }}_{i}\right]}^{2} \end{array}$$

(12) $$\begin{array}{cc} L_{total} & =L_{xy}+L_{wh}+L_{obj}+L_{class}+L_{\mathrm{orn}} \end{array}$$

where $L_{total}$, is the total loss minimized for oriented box regression.

#### 4.2.3. Ellipse Detection

**Figure 13:**
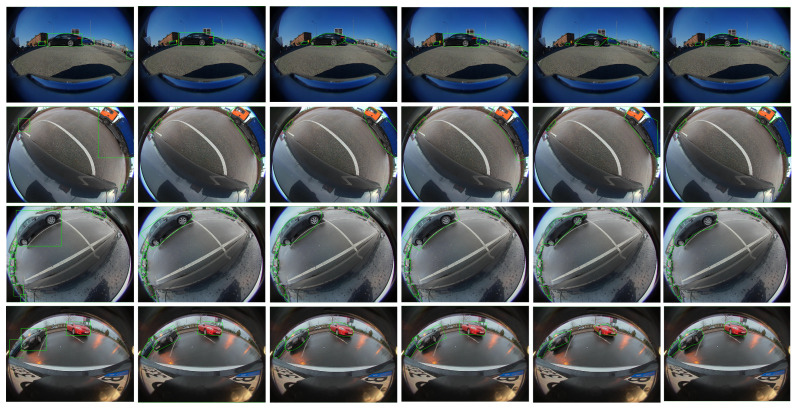
Qualitative results of the proposed model outputting different representations. Rows represent front, left, right, and rear camera images, respectively. From left to right: Bounding box, Oriented box, Ellipse, Curved box, 4-point polygon. 24-point polygon.

In this representation, we replace the bounding box by an ellipse, which has a better fit for vehicles, particularly with the fisheye distortion. As the vehicle tapers at its ends, the ellipse has a better fit than a bounding box, as seen in the black vehicles in the first and third rows in Figure 13. Ellipse regression is the same as oriented box regression. The only difference is in the output representation. Hence the loss function is also the same as oriented boxes loss.

### 4.3. Generic Polygon Representations

Polygon is a generic representation for any arbitrary shape and is typically used even for instance segmentation annotation. Thus, polygon output can be seen as a coarse segmentation. We discuss two standard representations of a polygon and propose a novel extension that improves accuracy.

**Figure 7:**
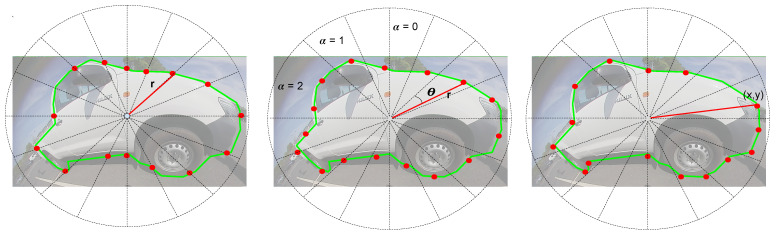
Generic Polygon Representations. (**Left**): Uniform angular sampling where the intersection of the polygon with the radial line is represented by one parameter per point (r). (**Middle**): Uniform contour sampling using L2 distance. It can be parameterized in polar coordinates using 3 parameters (*r*, θ, α). α denotes the number of polygon vertices within the sector, and it may be used to simplify the training. Alternatively, 2 parameters (x,y) can be used, as shown in the figure on the right. (**Right**): Variable step contour sampling. It is shown that the straight line at the bottom has fewer points than curved points such as the wheel. This representation allows us to maximize the utilization of vertices according to local curvature.

***Uniform Angular Sampling*** Our polar representation is quite similar to PolarMask [29] and PolyYOLO [31] approaches. As illustrated in Figure 7 (left), the full angle range of 360° is split into *N* equal parts, where *N* is the number of polygon vertices. Each polygon vertex is represented by the radial distance *r* from the centroid of the object. Uniform angular sampling removes the need for encoding the θ parameter. Polygon is finally represented by object center ($\hat{x}$, $\hat{y}$) and {$r_{i}$}.

***Uniform Perimeter Sampling*** In this representation, we divide the perimeter of the object contour equally to create *N* vertices. Thus the polygon is represented by a set of vertices {($x_{i}$, $y_{i}$)} using the centroid of the object as the origin. PolyYOLO [31] showed that it is better to learn polar representation of the vertices {($r_{i}$, $\theta_{i}$)} instead. They define a parameter α to denote the presence or absence of a vertex in a sector, as shown in Figure 7 (middle). We extend this parameter to be the count of vertices in the sector.

***Curvature-adaptive Perimeter Sampling*** The original curve in the object contour between two vertices gets approximated by a straight line in the polygon. For regions of high curvature, this is not a good approximation. Thus, we propose an adaptive sampling based on the curvature of the local contour. We distribute the vertices non-uniformly in order to represent the object contour best. Figure 7 (right) shows the effectiveness of this approach, where a larger number of vertices are used for higher curvature regions than straight lines, which can be represented by lesser vertices. We adopt the algorithm in [51] to detect the dominant points in a given curved shape, which best represents the object. Then we reduce the set of points using the algorithm in [52] to get the most representative simplified curves. This way, our polygon has dense points on the curved parts and sparse points on the straight parts, which maximizes the utilization of the predefined number of points per contour.

The polygon regression loss is given by

(13) $$\begin{array}{cc} L_{poly} & =\sum_{i=0}^{S^{2}}\sum_{j=0}^{B}\sum_{k=0}^{R}l_{ij}^{obj}{\left[r_{i,k}-{\hat{r}}_{i,k}\right]}^{2} \end{array}$$

(14) $$\begin{array}{cc} L_{total} & =L_{xy}+L_{wh}+L_{obj}+L_{class}+L_{\mathrm{poly}} \end{array}$$

The total loss is given by $L_{total}$, where R corresponds to the number of sampling points; each point is sampled with a step size of 360/R angle in polar coordinates, as shown in Figure 7. We used dense pixel-level annotations, and hence there is only one parameter needed to represent each polygon point in the polar coordinate system. It is similar to PolarMask. PolyYOLO, on the other hand, uses sparse polygon points (in red) and thus requires 3 parameters: *r*, θ, and α. Hence, the total required parameters for R sampling points are 3*R in the case of sparse polygon point-based annotations. The effect of different sampling rates w.r.t actual pixel-level annotation masks is presented in Table 1.

### 4.4. Distortion Aware Representation

Polygon is expensive both in terms of annotation costs and complex for usage in downstream tasks. Thus it is not a practical representation to use in practice. In this subsection, we attempt to derive an optimal representation of objects undergoing radial distortion in fisheye images, assuming a rectangular box is optimal for pinhole cameras.

#### 4.4.1. Banana Shaped Curvature in Fisheye Camera Projection

**Figure 8:**
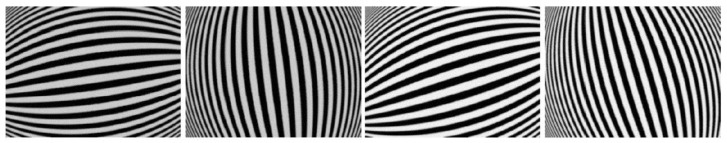
Illustration of banana shaped curvature via fisheye camera projection of black and white bands.

**Figure 9:**
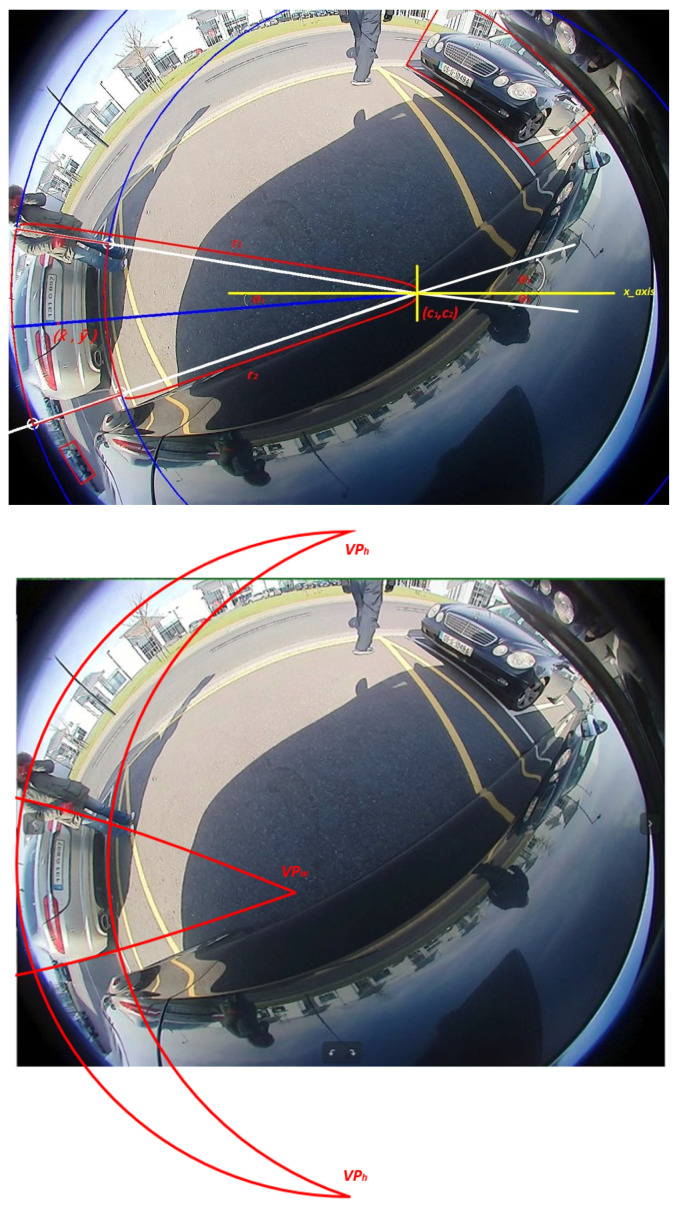
Illustration of our novel distortion-aware representations for the beige car on the left. (**Top**): Proposed curved bounding box using a circle with an arbitrary center ($c_{x},c_{y}$), outer radius $r_{1}$, inner radius $r_{2}$, and outer radius is illustrated. It captures the radial distortion and obtains a better footpoint. The center of the circle can be equivalently reparameterized using the object center ($\hat{x}$, $\hat{y}$), which lies on the central blue line. (**Bottom**): Vanishing points $VP_{w},VP_{h}$ are leveraged to constrain the curved bounding box with fewer degrees of freedom, from six to four. This is the natural representation of a box in fisheye images.

**Figure 14:**
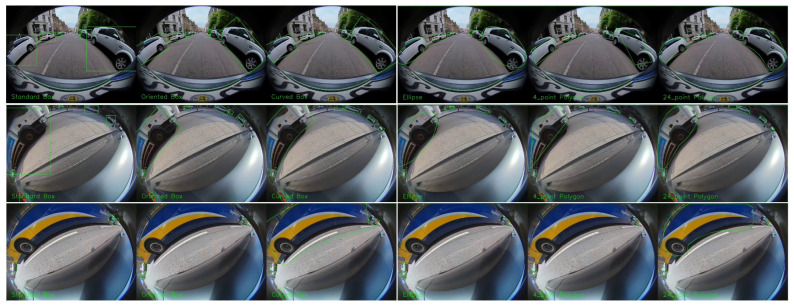
Failure mode analysis of the proposed model outputting different representations. Rows represent results on three sample images. From left to right: Bounding box, Oriented box, Curved box, Ellipse, 4-point polygon. 24-point polygon.

We project black and white bands on a 3D plane to a fisheye camera and visualize in Figure 8. The black bands appear to resemble banana-shaped curvature. This is only a loose analogy and not a mathematically precise representation. The red curve used to represent the vanishing point-based curved bounding box in Figure 9 also resembles banana-shaped curvature. It is also evident in the shape of the blue truck in Figure 14. We will use the more precise curved bounding box for the concrete representations we define in the next subsection. However, more research is required to find an optimal representation for the banana-shaped curvature as implied in the title ‘Lets Go Bananas’.

#### 4.4.2. Curved Bounding Box

In the pinhole camera with no distortion, a straight line in the scene is imaged as a straight line in the image. A straight line in the scene is imaged as a curved segment in the image for a fisheye image. The specific type of fisheye distortion determines the nature of the curved segment. The fisheye cameras from the dataset we used are well represented and calibrated using a 4th-order polynomial model for the fisheye distortion [12]. In this work, we consider the fourth-order polynomial model and the division model only. The reason is that the fourth-order polynomial model is provided by the data set that we use, and we examine the division model to understand if the use of circular arcs is valid under such fisheye projections.

In this case, the projection of a line onto the image can be described parametrically with complicated polynomial curves. Let us consider a much simpler model for the moment—a first-order polynomial (or equidistant) model of a fisheye camera. i.e., $r^{\prime }=a\theta$, where $r^{\prime }$ is the radius on the image plane, and θ is the angle of the incident ray against the optical axis. If we consider the parametric equation P(t) of a line in 3D Euclidean space:

(15) P(t)=Dt+Q

where $D=[D_{x},D_{y},D_{z}]$ is the direction vector of the line, and $Q=[Q_{x},Q_{y},Q_{z}]$ is a point through which the line passes. Hughes et al. [53] have shown that the projection onto a fisheye camera that adheres to equidistant distortion is described by:

(16) $$p^{\prime }\left(t\right)=\left[\begin{array}{c} D_{x}t+Q_{x}\\ D_{y}t+Q_{y} \end{array}\right]\frac{|p^{\prime }\left(t\right)|}{\left|p(t)\right|}$$

where

(17) $$\begin{array}{cc} \frac{|p^{\prime }\left(t\right)|}{\left|p(t)\right|} & =\frac{a\arctan\left(\frac{d_{xy}\left(t\right)}{D_{z}t+Q_{z}}\right)}{d_{xy}\left(t\right)} \end{array}$$

(18) $$\begin{array}{cc} d_{xy}\left(t\right) & =\sqrt{{\left(D_{x}t+Q_{x}\right)}^{2}+{\left(D_{y}t+Q_{y}\right)}^{2}} \end{array}$$

p(t) is the projected line in a pinhole camera, and $p^{\prime }\left(t\right)$ is the distorted image of the line in a fisheye camera.

This is a complex description of a straight line’s projection, especially considering we have ignored all but the first-order polynomial term. Therefore, it is highly desirable to describe straight lines’ projection using a more straightforward geometric shape.

Bräuer-Burchardt and Voss [54] show that if the first-order *division model* can accurately describe the fisheye distortion, then we may use circles in the image to model the projected straight lines. As a note, the division model is generalized in [55], though it loses the property of straight line to circular arc projection. We should then consider how well the division model fits with the 4th-order polynomial model. In [53], the authors adapt the division model slightly to include an additional scaling factor and prove that this does not impact the projection of line to a circle. They show that the division model is a correct replacement for the equidistant fisheye model. Here we repeat this test but compare the division model to the 4th-order polynomial. As can be seen, the division model can map to the 4th-order polynomial with a maximum of less than 1-pixel error. While this may not be accurate enough for applications in which sub-pixel error is desirable, it is sufficient for bounding box accuracy. Thus, we have mathematically shown that circular arcs are a good approximation for fisheye camera projection of lines in the 3D world. Please refer to [53] for more information on the mathematical details.

Therefore, we propose a novel curved bounding box representation using circular arcs. Parallel lines in the 3D world that project to parallel lines in a pinhole image, but they become circular arcs in a fisheye image. This is illustrated in Figure 9 (top), where the straight lines become circular arcs after projection. Figure 9 (top) illustrates the details of the curved bounding box. The blue line represents the axis, and the white lines intersect with the circles, creating starting and ending points of the polygon. This representation allows two sides of the box to be curved, giving the flexibility to adapt to image distortion in fisheye cameras. It can also specialize in an oriented bounding box when there is no distortion for the objects near the principal point.

We create an automatic process to generate the representation that takes an object contour as an input. First, we generate an oriented box from the output contour. We choose a point that lies on the oriented box’s axis line to represent a circle center. From the center, we create two circles intersecting with the corner points of the bounding box. We construct the polygon based on the two circles and the intersection points. To find the best circle center, we iterate over the axis line and choose the circle center that forms a polygon with the minimum IoU with the instance mask. The output polygon can be represented by 6 parameters, namely, ($c_{1}$, $c_{2}$, $r_{1}$, $r_{2}$, $\theta_{1}$, and $\theta_{2}$), representing the circle center, two radii, and angles of the start and end points of the polygon relative to the horizontal x-axis. By simple algebraic manipulation, we can re-parameterize the curved box using the object center ($\hat{x}$, $\hat{y}$) following a typical box representation instead of the center of the circle.

#### 4.4.3. Vanishing Point Guided Curved Box

In this subsection, we improve the proposed curved box in the previous subsection by leveraging vanishing point constraints of a fisheye camera, which leads to the reduction of degrees of freedom. A vanishing point is a geometric projective point where parallel lines in 3D space converge in 2D. Parallel lines meet at infinity in 3D, but the perspective projection in an image is finite. This is a commonly used cue in autonomous driving where lanes and road edges converging and can be used for consistency checks, calibration, etc. In the case of fisheye geometry, even finite parallel lines converge to a vanishing point due to the curvature of the distortion. Figure 9 (bottom) illustrates a sample set of vertical parallel lines associated with a bounding box in 3D converge to vanishing points $VP_{h}$, which is a set of two 2D points, one at the top and one at the bottom. Similarly, horizontal parallel lines converge to vanishing points $VP_{w}$. The point on the left is not shown for simplicity in drawing, as it lies far outside the image. Roughly, this resembles a vertical cut of a banana-shaped object.

Intuitively, a 2D box in 3D space gets projected into this curved box representation, and thus it is the optimal representation of a box representation in a fisheye image, as it needs only four parameters. However, it needs four additional points, namely $VP_{h}$ and $VP_{w}$, which are fixed for particular camera intrinsics but vary across cameras. We use a simple extension of the curved polygon loss to regress these parameters. Although this representation is theoretically optimal, we only implemented a naive representation in the YOLO framework. It can be better represented using other fisheye geometry representations such as spherical neural networks [56].

We outline the method to compute the curved box using vanishing points. We first project the horizon point in the image represented by the vector [0,0,1] in the camera coordinate system. The horizon point and the two horizontal vanishing points uniquely represent a circle, which we can call the horizon circle. Since the distance between the vanishing point and the object is fixed, we can draw a circle from the object center with a radius as half of the distance between both horizontal vanishing points of the object. We get the intersection of the new circle with the horizon circle to represent the new vanishing points. To project the “horizon point” on the image, we rotate the vector on the x-axis according to extrinsic camera parameters.

The object contour extreme points (left, top, right, and bottom) are obtained, and the vectors from camera space are projected to get the vanishing points. We then shift the horizontal vanishing points on the horizon circle to get the updated vanishing points. We then use the updated vanishing points to update the object’s extreme points by getting the max distance between each new shifted vanishing point and the object contour points. This algorithm has some corner cases that needed manual correction of the curved polygon estimates.

## 5. Experiments

All the box representations are systematically tested on the publicly available WoodScape dataset [12], which is the only fisheye camera surround view dataset for automated driving applications. Dataset details, metrics and evaluation criteria, and training details are presented in the following subsections.

### 5.1. Dataset and Evaluation Metrics

**Figure 10:**
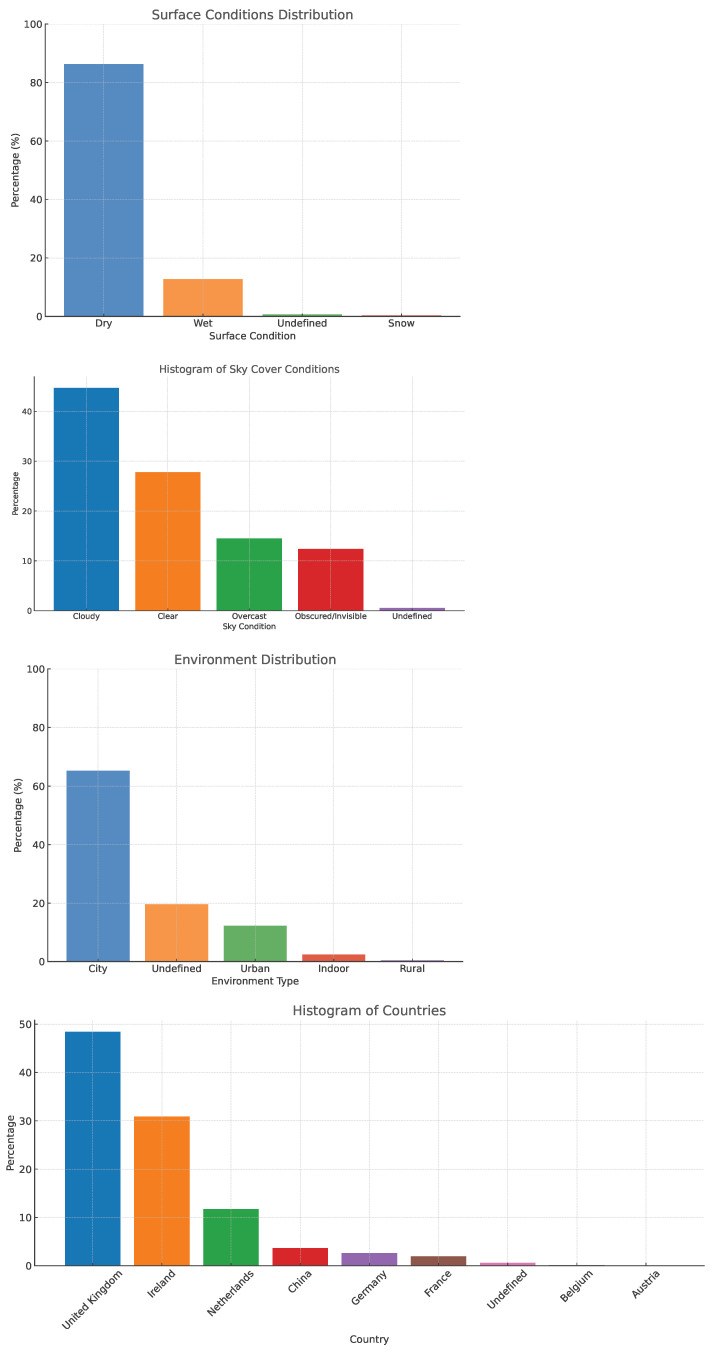
Exploratory Dataset Analysis of WoodScape object detection dataset.

We present an exploratory data analysis of the WoodScape dataset, which covers diverse scenarios [57]. Figure 10 shows the diversity of geographical, road surface condition, sky cover conditions, and road type of the dataset. The dataset comprises 10,000 images sampled roughly equally from the four views. The dataset comprises 4 classes, namely vehicles, pedestrians, bicyclists, and motorcyclists. Vehicles further have subclasses, namely cars and large vehicles (trucks/buses). The images are in RGB format with 1 MPx (MegaPixel) resolution and 190° horizontal FOV. The dataset is captured in several European countries and the USA. For our experiments, we used only the vehicles and pedestrian class, as the others were lower volumes. We divide our dataset into a 60-10-30 split and train all the models using the same setting.

The objective of this work is to study various representations of fisheye object detection. Conventional object detection algorithms evaluate their predictions against their ground truth, which is usually a bounding box. Unlike conventional evaluation, our first objective is to provide better representation than a conventional bounding box. Therefore, we first evaluate our representations against the most accurate representation of the object, the ground-truth instance segmentation mask. We report mIoU between a representation and the ground-truth instance mask. We could also report True Positive Rate (TPR) and False Positive Rate (FPR) by thresholding IoU for each object, but we find that mIOU (Mean Intersection over Union) is more informative.

Additionally, we qualitatively evaluate the representations in obtaining object intersection with the ground (footpoint). This is critical as it helps localize the object in the map and provide more accurate vehicle trajectory planning. Finally, we report model speed in terms of frames per second (fps) as we focus on real-time performance. The distortion is higher in side cameras compared to front and rear cameras. Thus, we provide our evaluation on each camera separately.

### 5.2. Training Details

Our models are trained on an input resolution of 544×288. A pre-trained ResNet18 model without classification layers is used as an encoder, and horizontal image flip is used as a data augmentation technique. All models are developed on PyTorch v1.4 [58]. Training, evaluation, and inference are performed on an NVIDIA GTX 1080Ti GPU. All models are trained for 80 epochs with early stopping criteria based on validation loss. Ranger optimizer [59] and one cycle learning rate scheduler [60] are used for optimization. Ranger uses gradient stabilization, which combines RAdam [61] and LookAhead [62] in one optimizer, resulting in stabilized training.

### 5.3. Results

#### 5.3.1. Finding Optimal Number of Polygon Vertices

**Figure 11:**
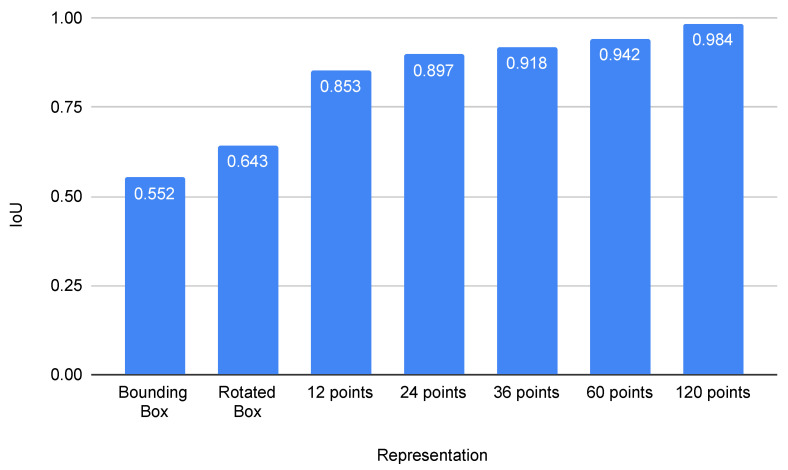
Analysis of the number of polygon vertices for representing the object’s contour. mIoU is calculated between the approximated polygon and ground truth instance segmentation mask.

Polygon is a more generic representation of complex object shapes that arise in fisheye images. We perform a study to understand the effect of the number of vertices parameter in a polygon. We use a uniform perimeter sampling method to vary the number of vertices and compare the IoU using instance segmentation as ground truth. The results are tabulated in Figure 11. A 24-sided polygon seems to provide a reasonable trade-off between the number of parameters and accuracy. Although a 120-sided polygon provides higher ground truth with far too many points, it will be challenging to learn this representation, and it will produce noisy overfitting. For the quantitative experiments, we fix the number of vertices to be 24 to represent each object. We observe no significant difference in fps due to increasing the number of vertices where our models run at 56 fps on a standard NVIDIA TitanX GPU. It is due to the utilization of YOLO [22] architecture, which performs the prediction at each grid cell in a parallel manner.

#### 5.3.2. Evaluation of Representation Capacity

Table 1 compares the performance of different representations using their ground truth fit relative to instance segmentation ground truth. This empirical metric is used to demonstrate the maximum performance a representation can achieve regardless of the model complexity. As expected, a 24-sided polygon achieves the highest mIoU, showing that it has the best representation capacity. Our proposed curvature-adaptive polygon achieves a 2.2% improvement over a uniform sampling polygon with the same vertices. Polygon annotation is relatively more expensive to collect, and it increases model complexity. Thus, it is still interesting to consider simpler bounding box representations. Compared to bounding box representation, oriented box representation is approximately 2.5–4% efficient for the side cameras and 1.3–2.3% for front cameras. Ellipse improves the efficiency further by an additional 2% for side cameras and 1–2% in front cameras.

#### 5.3.3. Standardized Evaluation Using Segmentation Masks

Table 2 illustrates the Vehicle and Pedestrian class breakdown scores. Vehicle detection performance is significantly higher, as there are more training samples available, and vehicles are rigid objects.

**Figure 12:**
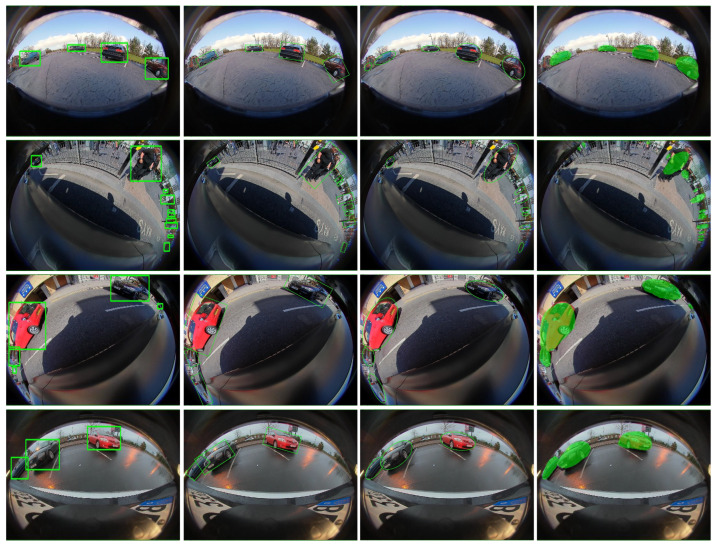
Visualization of generated annotations using WoodScape dataset that are used in Table 2. From left to right: Bounding box, Oriented box, Ellipse, and 24-point polygon (visualized as mask for better viewing).

Pedestrian detection performance is lower due to the non-rigid nature of pedestrians, as well as scenarios where pedestrians are very close and partially cropped, as shown in the second row of Figure 12. We also implemented two mAP scores where the output of the detector is compared to its respective ground truth and against instance segmentation annotation. As the ground truth representation is different, they are not comparable across representations. But it is interesting to note that the mAP scores are close to each other. When we compare with the instance segmentation annotation as ground truth, there is a large difference across the representations. As expected, the 24-sided polygon performs significantly better, but the gap is smaller compared to the representational capacity discussed in Table 1.

#### 5.3.4. Real-World Application Related Metrics

Complex polygon representation with 24 sides outperforms other representations in accuracy, as shown in Table 1. However, it is not practically suitable from a real-world application perspective due to significantly large additional costs incurred in annotation and training. In this subsection, we tabulate and discuss annotation times, inference times, and training times of the different representations in Table 3. The annotation times are averaged over 10–20 manual annotations to estimate the complexity. In the WoodScape dataset, these annotations are automatically generated from segmentation masks. Bounding box takes the least amount of time, and oriented box and ellipse take slightly more time to align the orientation. A curved box takes slightly more time. In contrast, a 24-sided polygon takes an order of magnitude more manual effort to annotate, particularly due to the variable spacing in the proposed polygon. Inference and training time are measured on an Nvidia TitanX GPU. The oriented box, ellipse, and curved box have slightly higher inference times. Polygon has a 15% increase in inference time, but it significantly increases the complexity of downstream tasks like tracking, prediction, and planning. Training times of bounding box, oriented box, and ellipse are the same, and the curved box has a slightly higher training time. Polygon model training is more than three times as long because of complex losses used in the proposed method. To summarize, polygon representation clearly outperforms other representations in terms of accuracy, but it is practically difficult to annotate large-scale real-world datasets and increases training and inference complexity. Thus, a curved bounding representation provides a suitable tradeoff.

#### 5.3.5. Ablation Study

Table 4 shows our studies on the methods to predict the orientation of the box or the ellipse efficiently. First, we train a model to regress over the box and its orientation. In the second experiment, orientation prediction is addressed as a classification problem instead of regression as a possible solution to the discontinuity problem. We divide the orientation range of 180° into 18 bins, where each bin represents 10°, making this an 18 class classification problem. During the inference, an acceptable error of ±5° for each box is considered. Using this classification strategy, we improve performance by 1.6%. We are formulating orientation of box or ellipse prediction as a classification model with IoU loss found to be superior in performance compared to direct regression. This has a 2.9% improvement in accuracy. Hence, we use this model as a standard representation for oriented box and ellipse prediction when comparing with other representations.

We used a simple ResNet18 encoder for computational efficiency reasons. To understand the effect of more modern attention-based encoders, we experiment with SAN-10 and SAN-19, which are relatively smaller than typical transformer backbones. We observe a significant improvement of 1.2 mAP. SAN-10 provided an improvement of 0.7 mAP. Given this result, improving network capacity can improve the performance further but will not degrade real-time performance. We then improved the complexity of the encoder by fusing two consecutive image encoder streams into an encoder of the same size. This can implicitly capture temporal cues, especially for moving objects. As we implemented a Siamese-style encoder, encoder computation is performed only once in steady state and increases the computational complexity only marginally. We observe a small improvement of 0.6 mAP score.

We then experiment with the proposed camera geometry tensor, which provides an inductive bias of the radial distortion. As observed in our previous study for depth estimation in SVDistNet [45], we observe a significant improvement of 1.4 mAP. This method is particularly useful for scaling up across a wide range of radial distortion geometries in a zero-shot manner without having to train for each new fisheye camera.

Finally, we study the effect of undistorting the radial distortion as shown in Figure 3. We make use of rectilinear and cylindrical corrections, which are commonly used. In the case of bounding boxes, the mAP score is significantly lower, as some regions in the periphery are lost, reducing the detection performance. Cylindrical rectification significantly improves over rectilinear undistortion by nearly 4 mAP points, as a significantly lesser field of view is lost. But it still suffers from resampling distortion effects and has lesser performance compared to baseline performance. Similar performance degradations can be seen for a 24-sided polygon.

#### 5.3.6. Quantitative Results on Proposed Curved Bounding Boxes

As discussed in the proposed section, a polygon is not a practical representation due to high annotation costs and high complexity for its usage in downstream tasks. Thus, we are motivated to design an optimal representation for the fisheye camera that is distortion-aware based on results in Table 1 and Table 2.

Table 5 demonstrates our prediction results for our proposed curved bounding box representations. Compared to the standard bounding box approach, the proposed curved box improves the performance only marginally by 0.7 points. However, the oriented box obtains an improvement of nearly 1 point over the curved box. The vanishing point-guided curved box obtains the best performance, outperforming the standard bounding box by 3 points. As we established in previous sections, the gap in performance between polygon and bounding box is high, and more research is needed to improve the performance of the proposed curved bounding box. Firstly, the algorithm for fitting the optimal curved box needs more exploration. Secondly, we only modified YOLO, which is designed fundamentally to output boxes, and more sophisticated methods that can natively model distortion, like spherical neural networks, might be a better model for the proposed curved bounding box representations.

#### 5.3.7. Qualitative Results

Figure 13 shows a visual evaluation of our proposed representations. Results show that the ellipse provides a decent, easy-to-learn representation with a minimum number of parameters and minimum occlusion with the background objects compared to the oriented box representation. Unlike boxes, it allows a minimal representation for the object due to the absence of corners, which avoids incorrect occlusion with free parking slots, for instance, as shown in Figure 13 (Bottom). Polygon representation provides higher accuracy in terms of IoU with instance mask. A four-point model provides high-accuracy predictions with small objects, as 4 points are sufficient to represent them. As the dataset has significant small objects that helped this representation to demonstrate good accuracy, and the same is shown in Table 1 and Table 4. Visually, large objects cannot be represented by a quadrilateral, as illustrated in Figure 13. A higher number of sampling points on the polygon results in higher performance. However, the predicted masks are still prone to deformation due to minor errors in each point’s localization.

#### 5.3.8. Failure Analysis

Qualitative results on randomly sampled test images are visualized in the video sequence shared at https://streamable.com/rgrp5h accessed on 11 June 2025. We have selected three frames in the video to discuss the main failure modes and visualized them in Figure 14. YOLO works by predicting the center of the object, which is the first step in all the object representations. Thus, in most cases, all the representations work similarly in terms of detection, but the more complex representations achieve much better bounded representation and mIoU scores. For example, the white car on the right in the first row image is detected by all the representations, but the standard bounding box has poor boundedness of the object, whereas the curved box and 24-sided polygon have good boundedness. In some cases, where the curvature of the box is very high, like the blue truck in the third row image, bounding box and ellipse representations fail to detect it, but the curved bounding box and polygon detect it. The main failure mode of all the representations is detection of small objects, which is illustrated by the missing detections in the middle of the top row image. In some cases of small objects, curved bounding boxes and polygons perform better, as shown by the detection of the small white car on the extreme right, which was missed by other representations. Partially visible objects like the truck on the left edge of the second row image are also challenging, but the model is able to detect them most of the time, as illustrated in this image. Side cameras on the left and right suffer more due to radial distortion of side vehicles, as shown in the third row image, where the large truck is distorted, resembling a banana shape. Curved box and polygon representations were able to detect this while the other representations failed to detect it. To summarize, polygon representation is consistently better than all the other representations. However, it is impractical due to high annotation costs, and the curved box provides a good trade-off, improving accuracy without increasing annotation costs.

**Figure 15:**
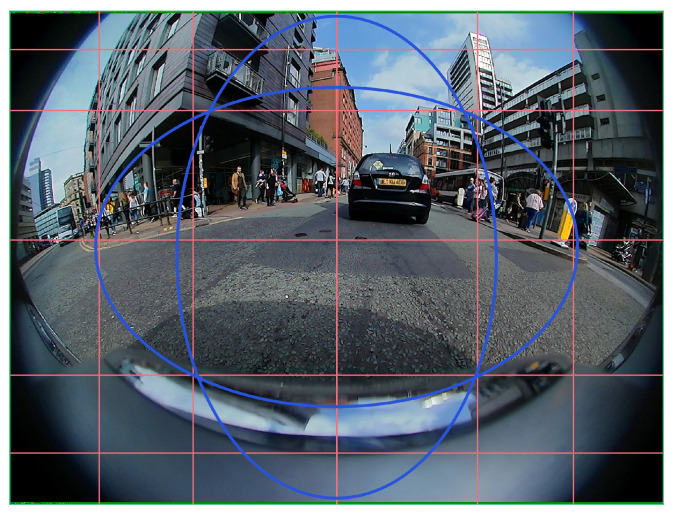
As a simple approximation, the area with the least distortion is defined as the union of the two ellipses, and the objects inside this central region are evaluated in central mAP calculation, while the objects outside this region are evaluated as peripheral mAP calculations.

**Zonal Metric:** Due to the high radial distortion at the periphery, it is important to understand the impact of fisheye image compression, particularly at the periphery of an image. We define the zonal metric such that the objects are evaluated in either *central accuracy* calculation or *peripheral accuracy* calculation. In the FOV of the camera image shown in Figure 15, the straight lines parallel to the y-axis act as the reference to indicate the curved nature of a straight building, and similarly, the straight lines parallel to the x-axis show the curvature of the windows, which would otherwise be straight in a pinhole camera image with no distortion. As the distortion increases towards the periphery of the image, we define the union of the two defined elliptical regions as the less distorted central region, while the rest of the area is considered the peripheral region. The elliptical region depends on the particular radial distortion of the camera lens, and this is a highly simplified method to classify less distorted and more distorted regions. We compare the zonal metric of the bounding box and curved box starting from Table 5. Central mIoU accuracies are 33.8 and 33.6, and peripheral accuracies are 30.4 and 32.8 for bounding box and curved box, respectively. The curved box has a slightly less central accuracy but outperforms in peripheral accuracy as expected.

## 6. Conclusions

In this work, we study the less explored problem of object detection on fisheye images. First, we demonstrate that due to strong radial distortions, the bounding box is not a good representation of object detection on fisheye images. We implemented a novel algorithm framework called FisheyeDetNet by extending YOLO to allow experimentation with different representations beyond the standard bounding boxes. First, we implemented basic representations like oriented bounding boxes and ellipses. Then we implemented a generic polygon, which serves only for comparison, as it is expensive to annotate and complex to use in downstream tasks. We observe that the representation capacity of a bounding box is highly suboptimal, trailing 35 points in mIoU compared to a 24-sided polygon. Thus we designed a novel distortion-aware bounding box representation called a curved box and refined it further using vanishing point constraints. The proposed method significantly improves the performance compared to standard and oriented bounding boxes by 3 and 1.6 mAP points, respectively. However, the gap is still large compared to a polygon representation. We hope that our work encourages further research in this area to find an optimal representation of objects in fisheye images. One limitation of this work is the naive extension of YOLO for new representations, and it would be a good direction to redesign the detector for complex curved boxes.

## Figures and Tables

**Table 1:** Model agnostic Evaluation of representation capacity of various representations. We estimate the best fit for each representation using ground truth instance segmentation and then compute mIoU to evaluate capacity. We also list the number of parameters used for each representation to provide a comparison of complexity. Bold refers to the best accuracy.

Representation	mIoU				mIoU	No. of Params
	**Front**	**Rear**	**Left**	**Right**		
Bounding Box	53.7	47.9	60.6	43.2	51.4	4
Oriented Box	55.0	50.2	64.8	45.9	53.9	5
Ellipse	56.5	51.7	66.5	47.5	55.5	5
24-sided Polygon (uniform)	85.0	84.9	83.9	83.8	84.4	48
24-sided Polygon (adaptive)	**87.2**	**87**	**86.2**	**86.1**	**86.6**	48

**Table 2:** Quantitative results of the proposed model on four representations. Representation vs. representation refers to the mAP metric computed with respect to ground truth with the same representation. In the case of representation vs. segmentation mask, instance segmentation annotation is used as a common representation to compare the different representations.

Experiment	Representation vs. Representation			Representation vs. Segmentation Mask		
	Vehicle	Pedestrian	mAP	Vehicle	Pedestrian	mAP
Bounding Box	**66.3**	**31.6**	**48.9**	51.3	31.5	41.4
Oriented Box	65.5	30.1	47.8	52.3	**31.9**	42.1
Ellipse	66	29	47.5	52.9	28.9	40.9
Polygon (24 points)	66.2	31.4	48.8	**67.6**	31.6	**49.6**

**Table 3:** Real-world Application Related Metrics. Curved Box metrics refer to both the variants.

Representation	Annotation Time (s)	Inference Time (fps)	Training Time (h)
Bounding Box	2.1	49	8
Oriented Box/Ellipse	3.1	52	8
Curved Box	3.5	52	9.5
24-sided Polygon	41.6	56	26

**Table 4:** Ablation study of configurations in the proposed architecture.

Configuration	mAP
Oriented Box	
Orientation regression	39
Orientation classification	40.6
Orientation classification + IoU loss	**41.9**
24-sided Polygon	
Uniform Angular	55.6
Uniform Perimeter	55.4
Adaptive Perimeter	**58.1**
Encoder Type	
ResNet18	58.1
SAN-10	58.8
SAN-19	**59.3**
Number of streams	
Single	58.1
Two stream	**58.7**
Camera Geometry Tensor	
Without CGT	58.1
With CGT	**59.5**
Effect of Undistortion for Bounding Box	
Rectilinear	39.8
Cylindrical	43.7
No undistortion	**45.4**
Effect of Undistortion for 24-polygon	
Rectilinear	52.2
Cylindrical	57.2
No undistortion	**58.1**

**Table 5:** Quantitative results of proposed model on different representations on our dataset. The experiments are performed on the best performing model according to ablation study parameters in Table 4.

Representation	IoU				mIoU
	Front	Rear	Left	Right	
Bounding Box	32.5	32.1	34.2	27.8	31.6
Oriented Box	33.9	33.5	37.2	30.1	33.2
Curved Box	33	32.7	35.4	28	32.3
Vanishing point guided Curved Box	**34.3**	**34.1**	**38.3**	**32.4**	**34.8**

## Data Availability

The dataset used in the experiments are available at https://drive.google.com/drive/folders/1ltj1QSNQJhThv8DVemM_l-G-GIH3JjMb.
